# Clinical Holistic Medicine: Metastatic Cancer

**DOI:** 10.1100/tsw.2004.189

**Published:** 2004-10-28

**Authors:** Søren Ventegodt, Elin Solheim, Mads E. Saunte, Mohammed Morad, Isack Kandel, Joav Merrick

**Affiliations:** ^1^ The Quality of Life Research Center, Teglgårdstræde 4-8, DK-1452 Copenhagen K, Denmark; ^2^ The Scandinavian Foundation for Holistic Medicine, Sandvika, Norway; ^3^ Division of Community Health, Ben Gurion University of the Negev, Beer-Sheva, Israel; ^4^ Faculty of Social Sciences, Department of Behavioral Sciences, Academic College of Judea and Samaria, Ariel, Israel; ^5^ National Institute of Child Health and Human Development, Office of the Medical Director, Division for Mental Retardation, Ministry of Social Affairs, Jerusalem and Zusman Child Development Center, Division of Pediatrics and Community Health, Ben Gurion University of the Negev, Beer-Sheva, Israel

**Keywords:** quality of life, QOL, philosophy, human development, holistic medicine, public health, life mission theory, purpose of life, character, coherence, spontaneous remission, cancer, metastasis, clinical practice, alternative and complementary medicine, Denmark, Israel

## Abstract

We believe that the consciousness-based/holistic medical toolbox has a serious additional offer to cancer patients and, as a consequence, designed a treatment for the patient with metastasized cancer. From a holistic perspective, cancer can be understood as a simple disturbance of the cells, arising from the tissue holding on to a trauma with strong emotional content. This is called “a blockage”, where the function of the cells is allocated from their original function in the tissue to a function of holding emotions. We hope to be able not only to improve the quality of life, but also to improve survival and in some cases even induce spontaneous remission of the metastasized cancer. This paper describes how work with a patient with metastasized cancer can be done in the holistic clinical practice in 14 days on an individual basis, helping the patient to recover her human character, purpose of life, coherence, and will to live, thus improving quality of life and possibly also survival time. The holistic therapeutic work includes (1) teaching existential theory, (2) working with life perspective and philosophy of life, (3) helping the patient to acknowledge the state of the disease and the feelings connected to it, and finally (4) getting the patient into the holistic state of healing: (a) feeling old repressed emotions, (b) understanding why she got sick from a holistic point of view, and finally (c) letting go of the negative beliefs and decisions that made her sick according to the holistic theory of nongenetic diseases. The theory of the human character, the quality of life theories, the holistic theory of cancer, the holistic process theory of healing, the theory of (Antonovsky) coherence, and the life mission theory are the most important theories for the patient to find hope and mobilize the will to fight the cancer and survive. The patient went through the following phases: (1) finding the purpose of life and hidden resources; (2) confronting denial; (3) taking responsibility for being very ill; (4) severe existential crises with no wish to live while still fighting; (5) integration of many repressed feelings and negative decisions thus rehabilitating character; (6) confronting lack of intimacy and trust in others and this way rehabilitating the ability to love; (7) rehabilitating the will to live, breaking through and falling in love with life; (8) assuming responsibility for the social relations; and sometimes (9) quality of life is improved radically with indications of spontaneous remission of the liver tumors.

## INTRODUCTION

Working with a patient with metastasized cancer and a very poor prognosis is the most challenging and difficult any physician can do. When the biomedical treatment has come to an end, the patient has been informed that the situation is hopeless, further treatment is not likely to help, and survival from the cancer is not possible, some patients prefer to turn to the physician with a holistic approach. In this situation, the holistic physician can do much for the patient by improving the quality of life (QOL) of the patient and thus help the patient to fight the cancer more vigorously and maybe even survive longer. It is a difficult situation with many pitfalls and it is extremely important not to make the situation worse for the patient by inducing depression, feelings of guilt, or by using the few resources the patient has on exercises that are not likely to help. It is also extremely important not to promise something that cannot be kept, not to give false hopes or illusions of survival.

In this paper, we will describe how we have been working with a patient with metastasized breast cancer, trying to avoid most of these pitfalls and succeeding in helping the patient to improve her quality of life. The purpose of our intervention in the holistic clinic is strongly inspired by the work of David Spiegel et al.[1], who helped women diagnosed with terminal cancer to improve their quality of life in the remaining part of their life. After 12 months, Spiegel and his co-workers demonstrated a significant improvement in the patients measured by various psychological tests: reduced pain, milder depression, and less anxiety, so we believe that quality of life can be improved for this group. Spiegel also showed one of the authors (SV) how he worked with these patients and how he supported the women in finding their internal and external resources for their struggle to improve the quality of life.

## IMPROVING COHERENCE, QUALITY OF LIFE, AND SURVIVAL

What we know about cancer is that it often develops over years, that dysplasia (change to the cells) is often an early stage, and carcinoma *in situ* (in the local place) is often found before the cancer metastasizes. We also know that the order of the cell as a whole, the tissue, and the organelles of the cells are often lost at the same time, making cancer a disease characterized by a multilevel structural breakdown. Quite surprisingly, cellular order is not just lost, sometimes it is also gained and the order of the body can reappear spontaneously. This is the famous picture of spontaneous remission of cancer[2]. How strange the concept must seem; the picture of spontaneous remission is rather well documented in the literature and the Danish researcher Ulrik Dige has shown that two of three patients going through such a spontaneous remission experience a spiritual awakening before the remission happens[3]. Let us define spontaneous remission of cancer as a remission that is not caused by a biomedical treatment normally considered powerful enough to cure it. When a patient is cured with holistic medicine, we call it “induced spontaneous remission”, which sounds like a paradox, but we find the meaning exact enough to use the phrase.

In our opinion, it seems that recovery of life purpose and human character is the factor most permanently connected to spontaneous remissions of cancer and it is therefore not surprising that working on recovery of the purpose of life and the human character[2] can lead to spontaneous remissions of cancer, which is the essence of our holistic, consciousness-oriented medicine. It seems in general that the cancer cells are disappearing by apoptosis, so a holistic cure for cancer is most likely to be successful if apoptosis can be induced[2]. (For every cell, there is a time to live and to die and there are two ways in which cells die: killed by injurious agents or they are induced to commit suicide. Programmed cell death is also called apoptosis.) It might be a surprise for many researchers who follow the typical biochemical way of thinking that induction of apoptosis seems quite possible and quite natural from a holistic medical perspective, as we believe that we are dealing with the same formative forces — the overall information system of the body[5,6,7,8,9,10,11,12] — in the body that induced apoptosis in embryonic life. If we radically can increase the coherence of the organism[13,14] and remove the disturbances that give the cells problems with communication, the level of information in the tissues can be radically improved due to holistic healing[15], and the apoptosis thus induced, as apoptosis presumably happens as a function of the cells realizing that it is not a natural and sound part of the body any more. To understand this line of thinking, let us review some of our work with holistic medicine and existential healing.

Many patients go to a holistic doctor in the hope of surviving the cancer, hoping that the cure will induce spontaneous remissions of cancer[16]. The common definition of spontaneous remission is “a complete or partial, temporary or permanent disappearance of all or at least some relevant parameters of a soundly diagnosed malignant disease without any medical treatment or with treatment that is considered inadequate to produce the resulting regression”[16]. A search for “spontaneous remission of cancer” on Medline (www.pubmed.gov) April 20$^{th}$ 2004 gave 5,458 hits, and it seems that spontaneous regression happens with almost all kinds of cancer[17], although it is reported far more often with some kinds of cancers[18,19,20,21,22,23]. A few years ago, there was a discussion if spontaneous remission of metastasized cancer really happened[24], as it might be a question of misdiagnosis[25]. Now the discussion is about the possible mechanisms and how it can be induced[26] — the ideal, optimal solution to a fast killing disease. The mechanism of spontaneous remission is not clear, but apoptosis seems to be a part of it[27]. There are many speculations as to what causes this apoptosis; some believe in immunological factors[28,29], like natural killing activity[30] or antibodies[31], while other speculate in a positive effect of fever[32], thyroid hormone stimulation[33], and surgery[34,35].

The incidence is also highly controversial. From 1900–1960, only 176 cases were reported, but some authors believe in numbers like 1:100.000[36], while other believe in a number 50 times higher for some types of cancers[37]. The Danish researcher Ulrik Dige[3] found over 40 cases without much effort, most not reported in the literature, indicating a ratio of 1:10.000 or even higher, so it seems that there is under-reporting of the spontaneous remissions. Dige[3] found that about two out of three patients had undergone some kind of spiritual awakening before the remission took place, indicating that the patient himself had an important role in the process of healing. It therefore seems that improving the quality of life could have something to do with survival and remission of breast cancer[1], but the psychological and existential elements in the process of spontaneous remission remains controversial[38,39]. The common definition seems to refer to the biomedical intervention only, as most of the spontaneous remissions (at least according to Dige[3]) seem to be induced by the patients themselves, alone or in cooperation with others. This is why we do not exclude the possibility of “induction of spontaneous remission of cancer”, even with the most terminal of patients, which literally is a contradiction[40]. At the same time, we always stress that the likelihood is small and that it is the patient, him/herself, who has to do the job of improving the quality of life and coherence in order to survive.

## CLINICAL HOLISTIC MEDICINE

The quality of life (QOL) theories[41,42,43] explain what quality of life is and how it can be developed. Interestingly, the most interesting resource in the person seems to be the depth of existence, meaning, or the purpose of life. The life mission theory[44,45,46,47,48,49] states that everybody has a purpose of life or huge talent. Happiness comes from living this purpose and succeeding in expressing the core talent in life. To do this, it is important to develop as a person into what is known as the natural condition, a condition where the person knows himself and uses all his efforts to achieve what is most important for him. The holistic process theory of healing[15,50,51,52] and the related quality of life theories[41,42,43] state that the return to the natural state of being is possible whenever the person gets the resources needed for the existential healing. The resources needed are "holding" in the dimensions of awareness, respect, care, acknowledgment, and acceptance with support and processing in the dimensions of feeling, understanding, and letting go of negative attitudes and beliefs. The precondition for holistic healing to take place is trust and the intention of the healing to take place. Existential healing is not a local healing of any tissue, but a healing of the wholeness of the person, making him much more resourceful, loving, and knowledgeable of himself and his own needs and wishes. In letting go of negative attitudes and beliefs, the person returns to a more responsible existential position and an improved quality of life. The philosophical change of the person healing is often a change towards preferring difficult problems and challenges, instead of avoiding difficulties in life[5,6,7,8,9,10,11,12,53]. The person who becomes happier and more resourceful often also becomes more healthy, more talented, and more able to function[54,55,56]. The above-mentioned theories are based on quantitative research in quality of life and health using new psychometric methodology[57,58,59,60,61,62,63].

## WHAT CAN BE ACHIEVED WITH A PATIENT WITH METASTASIZED CANCER?

The first and most important thing is to cooperate with the patient and help the patient to achieve what is possible in every single case. This is extremely difficult because the resources of the patient are often small, but if the will to live is strong, then there might be extraordinary resources in the patient. Therefore the assessment of the patient's resources and direction is the first thing to do and everything is dependent on this judgment. The second thing is to understand the patient's individual process of learning and transformation and especially what the patient is willing to give up in this process. Because the basic rule, if you want a fundamental renewal of your life, is the price of your new life for your old life. So if you will not let go of your old ways, values, perspectives, and habits, not much can be done for you. This willingness is in part related to the level of personal resources and in part related to the will to survive.

A general thing to learn from your cancer is that you can come closer to life[3,5,6,7,8,9,10,11,12,13,14,44,45,46,47,48,49,50]. You can change, you can move into life, you can conquer the meaning of life, understand its purpose, and step into character more fully — being yourself more. This strange process of "becoming yourself" is what holistic medicine is all about, because you are this wholeness and healing is to become your true, whole self. So dependent of the resources, the will to live and the willingness to "let go of the ego and surrender" and take learning and thus transform into a more true and whole person, there is very little or very much that a holistic physician can do for his patient.

The case in this paper is about a patient with intermediate resources and a modest will to live, but with an impressive willingness to take learning and to transform. The fast rehabilitation of the character and purpose of life made it possible to improve her quality of life in 14 days. We believe this patient was motivated for personal growth, but in no way exceptional. Her young age of 29 years and 7-year-old daughter was in the favor of the holistic treatment, helping us to mobilize her will to live.

## WORKING WITH A PATIENT WITH METASTASIZED CANCER

We worked with the patient in a team consisting of two male physicians (SV and MES) and a female physiotherapist (ES) with an intervention period of 2 weeks of 2-h sessions each time and therapy daily except for Sunday. We followed the four standard steps of holistic treatment[40,60,64,65,66,67,68,69,70,71,72,73,74,75,76]:

1. "Loving" the patient
2. Winning the trust of the patient
3. Getting permission to give the patient holding
4. Taking the patient into the holistic process of healing: feel, understand, and finally let go of your negative beliefs, attitudes, and decisions

The shadow side of the patient gave us fierce resistance[49], but we managed to establish and keep the alliance with her conscious self (her "white side"), helping her to identify the self-destructive patterns in her own (sub) consciousness. Little by little, she noticed the many negative beliefs that had been accumulating in her personal "philosophy of life" throughout her life. Letting go of them, she healed deep wounds in her existence and improved her quality of life radically — from a self-evaluated quality of life of "bad" to "very good" (from 4 to 1 on a Likert scale from 5 to 1, 5 being the worse). Quality of life measured by the QOL5 questionnaire raised from 3.8 to 2.3, which is very satisfying[51]. The patient went through the following phases in the course of the treatment:

- Finding her purpose of life and initiating the process by mobilizing hidden resources
- Confronting her complete denial
- Sudden realization of being very ill and taking responsibility
- Severe existential crises with no wish to live, while still fighting coming back to life
- Integration of many difficult feelings and negative decisions and rehabilitating her character
- Confronting the fundamental lack of intimacy and trust in others, rehabilitating her ability to love and be loved
- Rehabilitation of her will to live and BREAKTHROUGH feeling in love with life
- Returning home and assuming responsibility for her social relations
- Liver tumors at follow-up smaller [just before submission of this paper]

## CASE STORY

**Female, 29 years old, with breast cancer with numerous metastases to the liver:** Session (April 20, 2004): SV (leading physician) and MES (assisting physician) and ES (physiotherapist). The patient has been treated at the Norwegian Radium Hospital, Montebello, Oslo. Breast cancer detected April 29, 2003 with metastasis to the liver (ductal carcinoma, stage III). Received antihormone treatment, Zoladex injections 3.6 mg per month, and Femar 2.5 mg per day. Also chemotherapy, radiation therapy (25 times) with right breast and all lymph nodes related to it surgically removed. Growing liver metastases, three large and many small. We informed the patient that she enters a clinical research study with the purpose of improving her QOL, her survival, and if possible, inducing "spontaneous remission" of her cancer[2,77,78]. She is informed that we have made a search at Medline (www.pubmed.gov) today and found that the mean statistics for survival in her case is about 4.23 months[79] and spontaneous remission is extremely rare (this reference seems to be the one that most precisely describes the patient's situation; it says: factors that significantly predicted a poor prognosis on univariate analysis included symptomatic liver disease, deranged liver function tests, the presence of ascites, histological grade 3 disease at primary presentation, advanced age, oestrogen receptor [ER] negative tumours, carcinoembryonic antigen of over 1000 ng/ml and multiple versus single liver metastases; the patient has oestrogen receptor [ER] positive tumours, but multiple liver metastasis so we believe 4.23 month to be a fair prognosis based on this paper). We offer her an experimental treatment in a hopeless situation and we cannot promise her anything. She fully understood. Goal of treatment: Improvement of QOL measured by the QOL5 and QOL1 questionnaires (self-evaluated global QOL), improved survival, induction of spontaneous remission (induction of apoptosis, spontaneous cell death) of the cancer by rehabilitating her purpose of life, human character and will to live. Theory is given on: love, trust, holding, and processing together with the holistic process theory, the theory of evil and theory of character.

Session (April 20, 2004): Process on recovery of purpose of life and character[4]. The patient cooperates smoothly and we succeeded to find her purpose of life and character:

- Purpose of life: I am honest (may have to be upgraded)[46]
- Spiritual character: Truth (with law and will)[4]
- Mental character: Integrity[5]
- Physical character: Light/Humor (air) with clown, irony, neurotic, nerd, cynical (?), charm and firmness[5]

It made good sense to everybody, including the patient. This was a very fortunate opening of the therapy. EXERCISE: Act as the one you are!! EXERCISE (recapitulation) – with MES: Write your biography; start in the present, write about all incidents with strong emotional content - sexually, love, socially, family, work - related to all people you have known in your life. Make a complete list of all the people you have known. What happened? What did you feel? What did you decide? Goal: to make a timeline from conception until present time. (With MES) EXERCISE – with ES: Find out what prevents you from feeling trust and intimacy, and what feelings exist in the various organs and parts of your body. Plan: Improved QOL and health in 14 days, 4 sessions with MES, 4 session with ES, and 4 sessions with SV + MES + ES (a total of 24 h of therapy in 2 weeks). We arranged for the patient to live in a private apartment free for 14 days. This agreement was written as a "contract of treatment" signed by the patient and physician in charge. EXERCISE: Read the book *Consciousness-Based Medicine*[78](a popular book based on the papers[2,40,64,65,66,67,68,69,70,71,72,73,74,78,79,80,81, 82,83,84,85,86,87,88,89,90,91,92,93]); then read *The Power of Now*[94].

Session (April 21, 2004, MES): The patient has started writing the list of names; it is now three pages long and she is not done. She has not written anything else. First, a brief talk about the experience of being here, the different mindset, etc. She thinks it all makes sense, that it is "logical". Social information: The patient's mother converted to Jehovah's Witnesses as an adult, the patient's father did not want to. Today, she has good contact with her father, whom she visits several times per week. She feels fully accepted and loved by him. She works in the customer service of a transportation company. She has a 7-year-old daughter. The father takes care of the daughter half the time and it works out well. Afterwards, lying on the examination couch, she is told to try to think of a recent incident involving strong emotions. She mentions a phone conversation with her boyfriend the day before. He did not have time to talk; he was with his friends, which made the patient feel rejected. She feels sad and cries a little. She lacks his support every time she really needs it. The patient thinks for a long time before another incident dawns on her, which releases emotions: a brief message exchange with her mother around Easter time. "Mother has controlled me since I was little, I feel she only accepts me when she is in the mood – otherwise she does not." She does not really feel capable of making large decisions without her mother's approval. The patient is used to frequent sarcastic remarks from her mother, but she claims to have had a pretty good relationship with her mother last summer (impression: good when the patient acts appropriately). After this, she cannot remember any incidents involving strong emotions. She says she was kicked out of Jehovah's Witnesses 6 years ago. The course of events: she was going out with NN (also a Jehovah's Witness) and had premarital sex; she became a problem in the community. She had lots of feelings of guilt and ended up marrying him to clear her case, but changed to another community shortly after. Following was an incident of kissing another man at a party. Feelings of guilt drove her to confess, and she was kicked out of the community. Her husband was kicked out as well, when they discovered that he was snuffing tobacco and lost his driver's license after driving while intoxicated. This resulted in a very bad relationship with her mother. The patient joined the community again after her cancer diagnosis. The mother then took her back in. Brief talk afterwards: The patient still does not grasp the gravity of the situation, the time factor. I spell it out to her, and she reacts. (Impression: She is hard to get going. So far she has come in contact with feelings). Conference with SV: The patient must stick to working with her exercises. SV emphasizes this to the patient. I call the patient later that day to stress the importance of the exercises. Phone conversation at 4 pm: She says things are progressing slightly. She has a long list of names now and has started writing, but she doesn't think she experiences any feelings when writing about people and incidents involving negative feelings.

Session (April 22, 2004, SV with MES): The patient has worked on her biography, but she has absolutely no feelings associated with it. We get an image of the patient's "inner garbage bin" in the shape of a can that is just about to explode due to the rotten content, like the canned fermenting fish some Swedish people eat ("surstrømming"). We talk about the lid being glued to the can and that the patient will die when the can explodes. We agree to use today's session to loosen the lid so the patient can get in touch with her feelings. On the examination couch, SV press her ribs on the side first, then the side of her breastbone to solar plexus and then her heart. The patient is instructed to replace the physical pain, which is close to being unbearable with an emotional and existential pain, which is worse. The patient succeeds much better than expected. She starts out being angry and then says: "I don't like myself." She cries hard and the following sentences come out: "I hate you - I hate me - I detest myself - I disgust myself" - and finally, when I press a finger "into" the patient's brain: I am so unhappy". We talk about self worth, which according to the patient is at point –50 on a scale from –100 to +100. I think it is at –80. We talk about all the feelings she has of damaging herself, of being a failure, of feeling powerless and hopeless with self-hatred and anger. Many feelings emerge and the patient understands why she has become ill with cancer and what it takes to heal. She needs to acknowledge and integrate all these feelings, and when writing her biography, she needs to find the story behind all these feelings. EXERCISE: Use a roll of paper towels to let go of emerging negative sentences/essences. MES helps.

Session (April 22, 2004, MES): We work with "I do not like myself". Things are moving slowly and the patient must be pushed to get started. After 5 attempts there is finally a reaction and she cries for a long time. I try several times afterwards to work with the sentence "I detest myself", but it does not work. It does not "catch on" with her. I ask her to think about other words or sentences that may be a better fit. She quickly replies: "I am a bad person. I am evil. I am bad". She tries to let go of the first one. She immediately starts crying and does not have the energy to work on any more exercises. We have a brief conversation about the progress of her writing exercise. Impression: her motivation is falling and she has put the lid back on. I tell her this; she sees it and promises to work hard. According to the patient's wish, I remove 1 stitch and a drainage from her wisdom tooth in her left lower jaw. The patient has informed SV today that she is on hormone treatment (Femar) instituted by the clinic in Oslo.

Session (April 23, 2004, ES): On the examination couch, I work with the diaphragm, intercostally in the chest and back between ribs 9 and 12 and on quadratus lumborum. The pain is nearly unbearable, but she shifts between laughing and twitching in pain and "flails her arms". It is hard for her to enter the pure, physical pain and move to the emotional pain. "It hurts so much", the patient says. While I work on the psoas muscle, she starts thinking about the midwife, who completely wore her out when she delivered her daughter and she feels anger. At this time, I take her hand to support her and it really makes her sad (she cries). She acknowledges the fact that she did not receive or accept much support in her life. It seems to me as if support and care brings her in touch with her feelings more quickly than all the physical pain in the world, which is easier for her to ignore. She thinks about her daughter; feels sad again and cries: "I am a bad mother". She remains lying on the couch, while I physically support her and hold her. The sentence "if this does not work, I can just die" emerges and I reflect it back to her and tell her that it sounds as if she wishes to die. She becomes utterly sad and finds the sentence "I may as well die". The patient acknowledges that it is hard to allow herself to enjoy having me near her physically, and that I care for her. "It is not wise to think that having someone near you is good, because everybody disappears anyway," she says, feels sad and cries. We talk about the fact that she has been let down many times. We agree that she will continue to work on her biography and include all the times she has felt abandoned and let down, and to also work on these sentences: "It hurts so much" - "I am a bad mother" - "I may as well die" - "It isn't wise to think that having someone near you is good, because everybody disappears anyway". She feels pretty exhausted after the session and acknowledges that this is a painful emotional process. She can feel that she is entering the emotional pain, which she is actually afraid she will not survive. She understands the connection between acting dead emotionally and her illness, and that the emotional pain she feels right now makes her more alive.

Session (April 24, 2004, ES): She has been on the verge of crying all day, also on the bus on her way here. She read SV's book and says that it is not until now she believes that she can get well. She feels happy and sad at the same time. We work on the examination couch with iliacus/psoas, the diaphragm/solar plexus and above her pubic bone. Intense, almost unbearable physical pain. Negative sentences to let go of:

- I'm held down
- There is no room for me
- I am dizzy
- When I become ill, my mother loves me more
- I am willing to do anything to get my mother's love
- It is just physical pain
- I am in despair
- I do not get it (the feelings) out
- I am quite hopeless

I experience that I can do almost anything I want with her body and release the strongest pain without her reacting with anything but laughing and sweetness; "it hurts". I reflect back her indifference to her body, and her indifference in general. It is as if her body does not really concern her. No will to survive and live. These sentences then emerge:

- My body is not delicate
- I am clumsy
- My body is ridiculous
- I resign
- I give up
- I do not fix it
- I do not make anything work
- Nothing works
- I do not give a damn
- It does not work.

We talk about her being in touch with her resignation and resistance, and that it's hard for her to get in touch with her body and her feelings. I give her the exercise to let go of the sentences as many times as she can at night.

Session (April 25, 2004, MES): The patient felt down after body therapy yesterday. She did not think she could enter the feelings again. This was in hard contrast to the hope and the joy of life she experienced after having read the book *Philosophy of Life that Heals*[95], before she arrived in Copenhagen. Regarding the home exercises of "letting go": she finds it difficult and does not completely understand what good it does to let go of the feelings she has. Perhaps it is a linguistic issue. We discuss her work with "emptying the inner garbage bin", piece by piece. The patient feels that she is "in her head" too much, that she intellectualises. She has written 30 pages in her biography that are not chronological. She thinks she has got some clarity of some of her patterns and indicates that rejection is a recurring factor. She has learnt the technique of capturing the essence of the stories, which has sped up her work a lot. More memories keep emerging. Also names. I read a few of the incidents from her biography aloud, and we talk about the associated feelings. But she does not reach them. I then mention some incidents for her to tell me about in detail; she gets in touch with her feelings and cries a little. She finds it difficult to tell me these incidents several times, and I feel that we do not "get to the bottom" anywhere. The patient sees that she already very early on learned to put a lid on her feelings. Several people have described her as being "cold" and "strong" in highly emotional situations (including during her stay at the hospital, while being treated for cancer). She remembers an incident when she was 10 or 12 years old, where she cried in front of her mother and father (because their dog had been "put down" due to her mother's allergies and the patient had not been told). They huffed at her for not having discovered it sooner (apparently, it had happened a week earlier). She understands how she was not met with respect and love. Another incident emerges and she remembers her mother clearly indicating how much better the patient's friend was at writing than her. The patient "was never good enough" (in her mother's eyes) at school. Her father was more supportive. She tells me that she in certain situations pretends not to understand how she is offended; she acts stupid. "Are you stupid?" "No. I am smarter than they are", she says. The patient has described several incidents where she yells at and is tough on her daughter. She tells me that she finds the daughter to be "frustrating and irritating" when she cries and she often makes her daughter stop by being authoritative. She sees the parallel to how her own mother raised her. She feels sad. I compliment her for her good work on the biography and stress the importance of her having to work more intensely. Keep doing the let-go exercises.

Session (April 26, 2004, SV with MES and ES): We discuss the patient's trust in the treatment which is crucial SV offer her to get her money back (the half which is not yet used), if she already now knows that she does not have trust in the work we do. She has to choose and so she chooses to stay and work seriously, cooperate and form an alliance between her own "white side" and the white sides of MES, ES and SV. On the examination couch, we work with shame. MES takes care of the patient's safety, so she will not be in any more pain than she can endure. ES gives holding and SV process her through the rough pains of the past: "I am a pig who emerge nasty, ugly and disgusting" - "I am ashamed of my body and my sexuality; mostly my vagina and my clitoris", the patient says after an everlasting battle against her shame, which completely prevents her from saying anything for 20 min. "My labia are not filled with shame", she says; "I has been preoccupied with my clitoris and vagina since she was a child". EXERCISE: Place yourself in embarrassing positions at home and delve deeply into your shame; find out what it means and where it comes from. Write a journal of all the embarrassing and shameful incidents in your life. EXERCISE: Use a paper towel roll and let this go: "I am a pig - I am nasty - I am ugly - I am disgusting".

Session (April 27, 2004, ES supported by SV): In this session we use the sexological techniques called acceptance though touch[78]. We work on the examination couch with her shame. We work with her stomach and thighs and we talk about her having to take off her panties to get deeper into her feeling of shame. She does not feel like doing this at all, but she understands that it is necessary for her to process the shame that will emerge in her and she takes off her panties underneath the sheet. The sentence "I am far away" emerges, and she lets it go. She places her right hand over her genitals and ES places her hand on top of the patient's hand. There seems to be much shame associated to the support from the hand of ES. She fights against her shame by spreading her legs very slowly, until the soles of her feet touch and her knees drop to the side. There is much tension and pain in the adductor muscle group and in her hips. Slowly and gradually she removes her hand, so that ES's hand lies on top of and touches her labia, clitoris and the opening of her vagina. This makes her feel great shame and self-condemnation. Her sentences come out with the words:

- He must not see me
- It is nasty
- I am nasty
- It is disgusting/I am disgusting
- It is not right to do
- You do not do such a thing
- I become tired
- My legs hurt
- I cannot
- I am dirty
- I am gross
- I probably smell
- I am nasty and I like it
- I grieve for myself
- I am a nasty person, yikes…

- I want to throw up
- I have given up on myself
- I am crazy
- I am stupid

During the process, the patient remembers an incident as a little girl where she is standing naked in the bathroom with her mother, when her father enters. She feels fear and hides naked behind the door: “He must not see me”. She also remembers how her mother gave her clear instructions on how to wash herself thoroughly down below. Her exercise is to let all these sentences go using the paper towel roll. She has great pains in her hips, buttocks and loin. She gets a massage and care, which helps. But she feels “like a 90-year-old woman” in her body when she goes home. Later on the phone with SV, she is told what the positive sides of her character are. She has to explore this herself as well.

Session (April 29, 2004, SV): The patient shows no will to live today; she is completely negative, obstinate and contrary. She does not want to be here, does not feel like it. We talk about what is going on with her and reveal a death wish and an old feeling of wanting to take revenge on her parents, because she cannot have it her way. “Nothing happens”, the patient says. And I make her repeat it, until she remembers that her parents always fought, when she was about 9 years old. “Everybody is stupid”, she says, and “it is just nonsense”. “I do not think your tumours are growing anymore,” I say when asking about her illness. I have to disappoint her and tell her that as long as she does not want to live and continues to be so negative and against life, she should, in my opinion, not hope for recovery. She needs to find her will to live. That is a good project for the day. Say “I want to live” a 1,000 times”, I tell the patient. But this is extremely difficult for her. I can barely get her to do it. She puts her head between her legs and covers her face with her hands; she is in great pain. “I feel like sitting in a corner and doing like this,” the patient says and crosses her arms very tightly in front of her chest and face. She is only 2-3 years old now and it is impossible for her to live with all the people who are unfair and untrue around her. She fights, grieves, defeats herself, cries and says: “I want to live” in a more and more convincing way. “I want to live—eek—I want to live—I feel so stupid—I want to live...” EXERCISE: Go to the sea and yell, “I want to live” many times, until every fiber, cell and organ in your body wants to live. EXERCISE: She needs to let go of these sentences: “Nothing happens - Everybody is stupid – It is just nonsense - I do not feel like it - There, you see? (Revenge) - I do not want to - I do not get it?” I get her permission to make her journal public anonymously, in writing as well as verbally.

Session (April, 29, 2004, ES): The patient has been to the sea and tried to yell, “I want to live”, but she did not quite manage to set herself free. She is very tired and has given up on herself. It disturbed her that so many people were there to see her and hear her yell “I want to live”, and it deeply upset her that she could let that stop her from doing what she needs to do. My thought is that it resembles her experiences with her parents, but she cannot fully agree. We work on the examination couch with processing her negativity and lack of will to live. She needs to take a chance and use her will to choose life over death. I instruct her to go back to being a formless mass of energy, where she lets go of everything, all feelings, all thoughts, all form, and all ambitions… And when she reaches that point, I instruct her to remain in the energy, to feel its force and let it fill every cell of her body. Feel the energy and the force and tell yourself inside: “I want to live” and when you are ready and able to say it from all parts of you, from all your cells, you say it out loud as many times as you can. In the middle of the process, SV pops in and asks how we are doing and we are both even more moody and negative, but tell him that we are doing fine. We continue the process with an even stronger negativity. After about 20 minutes, she stops. “I am far away”, the patient says. She feels sad and in despair, because she cannot say “I want to live”. “I cannot fix it,” she then says and cries even more. It is my experience that she meets her negativity and processes it. We talk about the fact that the pain she feels is an old pain from a time early in her life, when she did not survive unless she changed herself. However, she is now an adult and receives the necessary support to change her decision, and she can use her will to choose life. She cries again and the sentence: “I do not want to die” emerges. She feels very sad and in deep despair. She repeats the sentence many times. “Then what do you want?” I ask. After a little while, the patient says, “I want to live”, and she repeats it 10-20 times. It sounds as if she means it. She cries and laughs. I cry and laugh with her. Afterwards she is both tired and happy, “like after a good orgasm or long lovemaking. I feel like I have just fallen in love,” the patient says. We discuss that she has to choose life over and over again, every time she encounters the choice. She has to be true to herself. EXERCISE: Make a positive flower, where the core is called “I want to live”, and every petal describes what makes living joyful to you—the opposite of negativity.

Session (April 30, 2004, SV with ES and MES): We talk about feelings and anger. She has to be emotionally honest in every situation. She has to pay attention to what she feels and what she can do. EXERCISE: to practice expressing her feelings in every situation with other people. We talk about what is ahead of her and I tell her how people normally feel and emotionally move up and down during a personal growth process. When they integrate old feelings they get new resources, which makes them able to get in touch with larger problems. When they integrate the new feelings, they again get new resources to get in touch with even larger problems. Often this continues for years. Plan: We are almost done for now. With ES: She can work with ES on expressing her anger; she can work with MES to get an overview of the past 14 days of progress she has just been through. With MES, who will make sure to get the patient’s own notes on record and to make an approximately 5 pages long resume with an introduction that briefly summarizes the patient’s progress. Continue doing your EXERCISES about acting who you are. I see myself in a different way - it’s good to be me. This is primarily thanks to ES’ session with the patient, where she learned to accept herself. The patient is informed that it is now up to her to stay on track and create a good life; she must put everything she has learned into practice. We formally review the result of the treatment with QOL5[96, for the calculations below see also this reference]: QOL5 at the beginning of the treatment: Q1-Q6: 3,4,6,3,4,4; QOL = (3.5+4+4)/3 = 3.8 (4 is bad). QOL5 at the termination of treatment today: Q1-Q6: 3,1,6,3,1,1; QOL = (2+4+1)/3 = 2.3 (2 is good). Self-evaluated QOL: Before: 4 (bad), after: 1 (very good). Difference: 1.5 scale steps of improvement on measured quality of life, 3 scale steps of improvement on self-evaluated quality of life. Overall a good result up until now. After she comes home, SV would like to be informed weekly via email about how she is doing. We discuss that she may possibly join the quality of life summer course and we will see what she needs by then. We must also communicate about how we can support her daughter.

Session (May 4, 2004, ES): “Where are you?” I ask the patient when we are about to start, 10 minutes late, because she has been talking on her cell phone. “I am at home”, the patient says. I confront the patient and tell her frankly that I am angry with her for wasting my time, which I have scheduled to help her and I am angry, because of her indifferent and arrogant attitude. I reflect that back to her and confront her with my anger. She responds in a patronizing and grumpy way and tells me to “get over it”. The patient describes that her body becomes timid - otherwise she would have walked away from this situation. “I feel like I am 7 years old and cannot talk back” (“I am not allowed” - “It’s not permitted” - “You don’t do such a thing”). On the examination couch we work with her timeline and the feeling of not being able to express her anger: I hold her stomach, chest and neck. 6-7 years old: She is picked on badly by her friend’s sister. She becomes angry and paralysed. Finally, she is chased off. 29 years old, she is treated for cellular toxicity: She is very tired and asks her father to take care of her daughter. But he will not, because he has to go get potatoes. She feels powerless and angry. She cannot say anything, because that will make him cross and sulky. 12 years old: Erika, the family dog has died, but she only finds out several days after the parents have secretly taken the dog away to be “put down”. She is sad and angry, runs out and bikes off far away. She feels rejected: Her reaction was unnecessary. She gets in touch with chaos when we follow her time line. It is hard for her to think of more incidents, although she knows there are many.

I apologize to her for having hurt her by getting angry with her and I respect the vulnerable state this causes her to enter. We follow the timeline again and search for incidents when her anger got dramatic consequences. I hold the front and back of her neck, chest and stomach. She does not remember any incidents, but she feels afraid. She is afraid to be abandoned. She is sad and feels pain/pressure in her chest and stomach. Sentences emerge: “When I am angry, you leave me” and “Everybody disappears when I am angry” - she LETS GO of this under much resistance. She feels sad and cries. We talk about her having to let go of this sentence many times, so she can loose her resistance to being angry. Contain her anger of being rejected, as opposed to repressing it. Contain her anger instead of enclosing it with her body. Dare to be rejected, but not reject herself. Choose to live. We look at her human character and purpose of life in order to review what it means to act out your character and discuss how to form an alliance with the good and positive in your character in choosing to live. This is the last treatment she gets.

Note (October 4$^{th}$ , 2004 and DAY 150 [SV]): Concluding remark. She is doing well. I have received several sets of ultrasound pictures from the University Clinic in Oslo showing in successive ultrasound examinations that her lever metastasis are disappearing (compare her diary below). Now there are only a few tumours left in her liver. She has attended our 5-day summer course on quality of life and she is still working on improving her quality of life further. It seems thus that her development is positive and stable.

## DIARY OF THE PATIENT

DAY 1: First session with Søren (SV), where he introduces me to Elin (ES) and Mads (MES), who will apparently also work with me. We talk a little, and then he rearranges the chairs, so that Elin (ES) comes to sit right next to me, really close. I get hot, and don’t like it! Søren asks what happens and I ask him if this is necessary – she crosses my barriers, I don’t want her so close! I move away a little and he asks me if I know what I am doing? Am I aware, that I am rejecting the trust she tries to show me? I get embarrassed, put out...he is right! What is wrong with me? So I move my chair back, and stay there for the rest of the session. I am told to write my autobiography, from this day backwards, to my birth. Oops! And me that cannot remember my childhood!? We find my life mission: I am honest. It doesn’t take them many minutes to describe the exact person that I am…scary.

DAY 2: Session with Mads: I was lying on a bench like at a psychologist, talking about emotional episodes. Cried some. Must start with my biography now. Søren (SV) and Mads (MES) are pushing me hard. Mads (MES) is concerned that I haven’t realised the severity of my situation, how little time I have left. I go home to get started. First the namelist….oh my god, 300 names so far, and there are still many that I have forgotten. Spend two hours on the biography, not easy. Feelings and tears coming out, and then it is over. Cannot remember any more episodes, feel completely cold. Worries me.

DAY 3: Session with Søren and Mads: I say I am worried about the writing, no more feelings. Søren explains that he will help me with this today. Asks me to undress to my underwear…I resist. Undress? On the bench I lie with a sheet over me. Søren asks me if there is anywhere he may not touch me. He wants to touch me EVERYWHERE????? Well, I must trust him, so ok. Mads is also to touch me. Ok. Søren finds places on my chest where he applies pressure, says that this is where I "hide" feelings…..it hurts like hell! He tells me to transfer the pain into emotions and let them out….transfer??? He says I must feel, not think! At the same time he talks to me about the negative stuff I may have hidden here. He asks Mads to hold my hand for support. THEN the emotions appear! I cry a lot. Søren helps me to verbalise them, asks me who I hate….I hate ME!!! I cry, I feel the pressure from his hands and it is very painful. He moves his hands to over my heart. Terribly painful, even worse than before! I feel very unhappy now. Cannot stop crying. He moves his hands to my belly, where the self-value emotions apparently lie. Does not hurt physically, I cry less, but tears keep rolling down my cheeks… He asks me what score I would give myself, in value, on a scale from –100 to +100, where 0 is neutral. I say –50. Søren says –80, I cry and cry….I realise that I simply don't like myself! I see that Søren(SV) has helped me remove the lid on my "internal garbage bin". I had it glued on with superglue. Søren gives me an exercise: to say the negative sentences out loud, while I hold something in my hand and squeeze it as hard as I can, thereby transferring the sentences to it, and at the same time touch my forehead in the middle just above the eyebrows, to make contact to my spiritual side. Say it out loud to myself so I feel the emotion, and then let it go. I am to do this every morning and evening, repeat the negative sentences…"I hate myself, I puke at myself, I despise myself, I don't like myself, I am a bad person", etc. etc. After a while I suppose more sentences will come…the point is to *feel* the emotion, then squeeze it into something else, and then let go of it. Søren goes and Mads keeps working with me on this. He is not merciful…. I cry…and finally can no more. I get dressed, we small talk a little and borrow their computer to check my email. Mads comes back with an earnest expression…he worries that I am not fully serious about this. That I have already put the lid back on. I can feel that it is true. It is NO problem to put it back on… Had tea with Søren in the evening…. I appear to be a "difficult" patient. He says I am very ignorant and distant to the world. That I am very neurotic and controlled by feelings that I deny. I don't realise that I am very ill and will die…SOON if I don't let go of my old patterns. He said he was surprised at how hard he had to work on me today…that I am very cold.

DAY 4: Søren says I must pay the fee now, partly symbolic, so I don't consider these days a "testing period" for effect. If I pay now, I get more hungry to get value for money…I get the idea… Session with Elin: We talk a little, she asks me to lie on the bench. She uses much the same technique as Søren…It really hurts. She applies pressure and squeezes my back, for a long time. Then she says it feels like I manage to leave my body. I think she is right, for I can only feel physical pain. After a while she takes my hand….THAT'S when my feelings burst out, and I cry. I admit that if this doesn't work, I might as well die! I am deeply unhappy, a poor mother, a bad human being…. Get these expressions onto my list of sentences to let go of. I am very tired….. and sad. And very fearful that I will fail to let go of these feelings. New talk with Søren in the evening: Today he talks about my friends and relatives that will fight the new "me" when I get back home. That I must find new friends that share my view of philosophy of life and I see what he means. A good friend from home had called me, cried and was angry, didn't understand why I am going through with this, wanted me to demand a second round of chemotherapy instead. I felt no support from him. Søren says it is important I stay away from him for a while. Only share what is happening with those who can support and believe in what I am doing. Take use of the friends that give energy, not the others. And me thinking that hell was over once I got back home….

DAY 5: Started reading Søren's book on *Philosophy of Life that Heals*[95] and had an enlightening experience. He actually knows what he is talking about. I see my body as a bunch of cells…that are just like single individuals. Strange. Feel like I am high….happy and sad at the same time….I am going to get well!!!

Session with Elin: I am initially happy and chatting stupidly, feeling certain things will go the right way…but no. Elin squeezes my belly and pelvis. Hurts a lot, but no emotions. Can't "disconnect" my head. Get disappointed….am I really this cold? Elin says I am one of the coarsest, most ignorant patients she has had. That everything impacts my body, but not *me*. Scary. Find many new sentences to work with tonight….got to get going! I leave, sad and disappointed with myself.

DAY 6: Tea with Søren: He is mad at me. Feels that I am hiding behind a facade of being stupid, while really being quite intuitive and intelligent….who me??? I think he is right. It is easiest not to make so much of myself, not be in the way. But Søren says that life is not about being in other's way, the point is not that others like you, but that I can do something of value to others. Why is it so enormously important for me to be liked?? I think about my job, that I do nothing meaningful to others there.. Søren says I must stop trying to understand everything with my head, that it is impossible….and I know that this is a great part of my problem. I DON'T UNDERSTAND!!!! Søren says that even if I think things are not progressing, they are. I just don't realise it. Session with Mads: Things go much easier today. He reads up some of the situations from my past, we talk about them. He asks me to tell the story of my cancer, the treatment, etc. I realise how distant I have been to it all. How much I have abused my body. I feel a deep sorrow and cry. That I have been so stupid… I also realise that I haven't had much support in my life. No wonder I have learnt to repress my feelings! It is tragic to see my patterns, and understand how "destroyed" I am.

DAY 7: Session with Søren, Elin and Mads: Søren starts by apologizing for being a little hard on me, that he has fought a lot with my doubts, etc. He sees that he doesn't love me enough. I think: "of course he can't do that, I don't let him in…" I cry. He says, he has considered giving me half of the money back, and send me home. I must answer if I want this. I DON'T! He says ok, but then I must decide to believe in that he can help me. I do that, realising that my defences have been producing my doubts. On the bench, Elin and Mads give support. Søren talks and finds points to press on. Asks me to say: "I am a pig" (a dirty person). It is VERY hard for me to say it; I just lie there, laughing. After a while I understand what he is aiming at…I cry and cry. He talks of shame, guilt, sexuality and food. Speaks a lot of shame over my sexuality and wants me to name the body parts that I am ashamed of….very hard to say it out loud. Finally he says we can take another approach if I like….ok. He asks me to spread my legs a little…WHAT?? Spread my legs?? Is he going to touch me there??? No thanks! I must say it instead. He helps me find the words, I say them….phew! That was hard! This was an important lesson for me. I get a good hug from Søren before I go, and feel much more confident in him now…good! I am given as an exercise: To find sexual positions that I am ashamed of…eh? I feel there is no such position for me, but Søren figures that I haven't tried hard enough…hmmm.

DAY 8: Feel very tired. Last evening was tiresome, everybody called me. Had no peace…realised I have to tell everybody that I need peace, that I will contact them if I need to talk. They don't understand what I am doing anyway. Session with Elin and Søren: Today they will work on my shame of my sexuality, and Elin suggests that I take off my underpants and that they will touch me. I feel my tummy tighten, I get embarrassed and worried. Start to cry. I don't want to, one can't do that! I finally take them off. Søren asks me to put my hand on my pelvis. I do it, and Elin puts her hand on top of mine. Then he asks me to spread my legs. My legs lock themselves… spread my legs??? Tears roll and roll down my cheeks and I fight a huge fight with myself. Suddenly Søren asks me where I am…I have fled! To the universe, somewhere, far away! I let go of the sentence: "I am far away", and try to focus. Søren has another patient, must go, and leave me with Elin. She asks me to spread my legs, very slowly, stop along the way, and sense the emotions that come to me. It is a big fight, lots of bad feelings and shame. And I realise that I flee within a moment, can hardly feel I am doing it. It happens a lot, I have to work hard to be present. It hurts, but is at the same time strange to experience. Terrifying to see that I have repressed so much shit. Elin asks me if I am ready to remove my hand. What? I remove it slowly, and finally her hand is on my pelvis. I am ashamed to realise that I enjoy it… Yuk! She asks me which emotions come up, and at one point I feel sick. I am disgusting and sick and shake my head and body (and I do it again now as I am writing...). Elin asks me how old I think I am. I don't know, cannot see that these are feelings I had as a child. Afterwards she touches me even more closely, I find it disgusting. What if I am smelly? Now she gets it on her fingers, I get sick again. It is really a very tiresome session; I have a headache at the end of it. Elin gives me some massage for my sore back and thigh, they just hurt more and more. She thinks the pain is also due to the process I am in. I get a sheet of paper with 18 sentences to let go of at home…phew! I ask Elin about the purpose, the effect of this process. She explains that it is to acknowledge what burden the cells are carrying. Finally an answer that I understand!! I have to let go in order to help my cells! Of course! Leave with a deep sensation of sorrow. Work hard on letting go in the evening. My body feels heavy and sad. Very tired, headache. I almost feel sick while doing it, my head is shaking. Crazy. Look forward to becoming a "new" person, the real me, for this is gruesome to go through. I realise what Søren meant by having to "die" to become the "real me". If I survive this, I will survive ANYTHING!!!!

DAY 9: Session with Mads: We work on the shame-part. He read out from my notes on shameful experiences. Not so much feelings today, but on having to tell the same story several times I realise that more and more details come to me. Strange. We find out that the shameful sexual episodes I have written about actually weren't shameful…so where does the shame come from?? I cannot remember any sexual assault during childhood, but when Mads asks me if I have been raped as a grown-up, I jolt. But have no memory of this either, so I don't understand that reaction. The brain is funny…. I go home and feel completely at loss. Cannot remember a single episode, everything goes very slow. Try to let go of some sentences, but I feel nothing. I give up, have a shower.

DAY 10: Session with Søren: He says we must get my will going! He asks if I think my cancer has stopped growing. I say I think so, he says it probably hasn't! Because I lack the will to live! He asks me to say out loud: "I want to live!" I have great difficulty in saying it…start to cry and want to hide myself. He asks me to go out to the ocean and scream out loud that I want to live…hmmm…ok. I find a suitable place on the beach and manage to say it quite loud, but worry that someone will hear me. I know it is stupid, but cannot overcome that fear, feel very silly. Work on it for an hour; feel that I am going home without having accomplished what I was supposed to. Disappointed and tired. Session with Elin(ES): I am frustrated. Why was I so worried that people would hear me, who do I connect these people with? I don't know. I lie on the bench, Elin wants me to sense all my emotions, and let go of them, feel that I am only mass and energy….and feel that I want to live with every cell in my body, and then say it out loud. Søren knocks on the door, interrupting us, and I try again…but cannot. Get very desperate, Elin says I must use my will…Tears roll and I want to escape… Elin explains that this was how it was for me when I was a child, with no support, but that I now have the support I need. I start to cry a lot, and Elin cries with me. Finally it comes to me: "*I don't want to die*!!!" Elin asks me what I want, and I say: "**I want to live!!!**" I cry and cry and repeat that I want to live. Suddenly, I start to laugh, and we both laugh a lot. Feels good, I feel like in love, high on life, call it what you like, but I am very happy! Tired, but very happy! I am given an exercise: To draw my positive flower. I walk off feeling that I have lots of energy, merely floating down the street. Imagine, *I want to live*!!! Great! I think about all that I want to do in the future. I want to live!!!!!!!

DAY 11: Session with Søren, Elin and Mads: Still high on life, I am bubbling! Søren says that he had planned to send me back in time to my foetal age, but is reluctant, since I am so delightedly happy…We finally agree to get me on the bench, and he will try to bring out my anger…he says anger sets itself in the liver! Oops! Makes sense! I know that I can explode if I get really mad. Elin and Mads support me. Søren pushes and squeezes so hard I think my ribs will break, but I cannot get angry, it just hurts really badly!

He tries to make me call him names, to growl and scream, but I can't. Just laugh and feel really stupid. But I see my problem. I have always repressed my anger, because one just doesn't get mad at people, and the few times when I have, it has taken off completely…it scares me a lot to think about having to tell people off when I get angry… Now Elin switches with me, gets on the bench, and growls and screams "I hate you" to Søren. But it does not help to make me yell at him…sigh! I get dressed, and we talk a little. I sign the papers on allowing them to use my case in an article. And then it hits me, are we finished??? Is this all? Help!!! I walk home and feel that everything is chaos! How will I survive at home? If I start telling people off, I will be all alone, etc. I feel fear of going home. FEAR!

DAY 12: Session with Mads: My body feels strange, bad, I am sweating even though I haven't been running, yuk! This session doesn't make sense to me, so we make a new appointment for tomorrow. Back home, in bed, I am suddenly hit by the fear that I used to have for demons during my childhood. I fall asleep and dream that something or somebody tries to tear off my sleeping quilt, and wants to hurt me, scare me. I get terrified and breathe heavily, like paralysed…it is all very lifelike…painful…I am very afraid!

DAY 13: Wake up, very tired. Feel alone and afraid. I burst into tears and cry for a long time. Cry myself back into sleep. When I wake up again things feel lighter and Elin joins me for a trip to Tivoli. We have a good time and talk a lot. Sleep well this night.

DAY 14: The biggest fear is over, I know I can succeed! Good mood today. Talk with Mads; we go through the whole therapy period. I realise that I have changed, and how important it is to me not to fall back into old patterns when I get back home. It will be a big fight, for I understand how I have been hiding instead of living! Locking up all emotions, not feeling, not sensing! Session with Elin: I arrive 15 minutes late. Elin tells me off for being late and I feel my chest tightening. She says I am "pissing on" the time she has reserved for me. I get very ashamed and apologize. But she doesn't give up and I feel I get angry. This will have to do! At the same time I suspect that she is trying to "trigger" my emotions and I must say she succeeds. I get moody and think "get over it", and Elin finds me condescending…I recognise the pattern; this is what I have always done! But I can't just answer back? One doesn't do that! We discuss it, and I realise that I have learnt this pattern early. One must be polite and never step on someone's feelings…but what about *my* feelings??? Hmm… I get on the bench, and Elin asks me to visualise myself from today and backwards, to my birth. Not think, just close my eyes and see what happens. The earliest episode with anger….I remember being told off by a girl after I had told her little sister off. She scolds med completely, and I get paralysed, cannot say a word, just feel my anger growing. In the end she chases me, and I just walk away. I guess I was about 7 years old. Elin asks me to close my eyes again, and I remember a time when I was very tired, needed support to look after my daughter for a while. I asked dad for help, but he wouldn't help me, he was about to go with someone else to pick up potatoes…. I got furious. Potatoes???!!!! But I didn't say anything, because then dad would become moody (I feel my tummy tightening now…)! I also remembered when my parents had my dog put down without telling me, and how angry I got when they just laughed at my sorrow…and I just took off on my bike… Close my eyes again, I am now to find an episode where I got angry, and it had dramatic consequences for me. I remember nothing, everything is just chaos! My belly is singeing, think it is the lunch I had, only it is a strange burning sensation. Elin says it is probably the fear I am feeling. Of course! Elin then asks me how I feel about the sentence: "When I get angry, you leave me". I start to cry. She asks me then to let go of it, and I feel great resistance. But finally succeed. We are done, and I am worn out. Feel the fear of going home in my belly. I think I had never gone home if it wasn't for my daughter… I sit, trying to write about what happened today with Elin, but cannot. It hurts too much. I understand how I am "saving" myself by not letting my anger out. A friend calls me, and I get annoyed with her, but do not express it. Why??? I feel stupid and finally call her back to tell her off, realising that this was an excellent opportunity to practice….Phew! It went well.

DAY 15: Mads calls me, says he needs the rest of my diary before I go back home today. My belly tightens again! I guess I had hoped it could wait a few days…? I am a big girl. Imagine that this should be so hard! I see how I am trying to hide from myself, cheat on myself. Ok, I'd better get going…

DAY 41 (e-mail): Today I had an ultrasound test of my liver and remember that the case record said there were many small tumours here? They are GONE!!!!!!!! ☺ ☺ ☺ I am so happy ☺ ☺ ☺ There is one big and one much smaller now; I will get the proper test result in a month! ☺ I like you so much☺ Have a nice weekend☺

DAY 121 (e-mail): Good news – another tumour has gone!!! I was at ultrasound today and now they could only find two tumours… formally this is only a temporary answer (until I meet with the physician), but they said it looks good and positive! I am so happy….. and gratefull for your help, dear Søren and Elin! Big hugs and lots of love ☺

## DISCUSSION

Improving the quality of life can be done in a few days or weeks[61,62,63] by the recovery of character[4], purpose of life[46] and will to live[53,54,55,56,57,58,59,60]. It is not known if the effect of this treatment on quality of life is permanent and we see that emotions will be unstable for month or even years afterwards[66]. Our patient has both biomedical and holistic physicians and this can give her problems because the two paradigms understand life, healing, and the cancer disease very differently. The physicians are therefore likely to give her different and conflicting advice. If she follows one advice the other might judge and condemn this treatment. In this situation, biomedicine did not have much more to offer, so there were no big ethical issues[2]. In many cases like the present, the combination of biomedical and holistic treatment is very simple and without any problems at all; when the breast cancer had metastasized to the lung or liver, there is no effective known cure and the biomedical physician will usually not hesitate to accept that the patients try any kind of alternative treatment.

It is important to say that our consciousness-based medicine has not been clinically tested at this moment. We believe that all physicians are ethically obliged[68,69,70,71] to take medical science a step further and develop new treatments, where the old ones have failed or are insufficient. We believe that the new medicine can help cancer patients subjectively; as we have already seen much good happen for the patients using it with a variety of diseases.

We are in the beginning of using this method and we do not know very much about its biological effects or side effects and we will undoubtedly make some mistakes while experimenting in spite of our very best efforts. As we see it, the holistic treatment of patients with metastasized cancer has very few and very limited negative side effects, but instead a positive impact on the quality of life, general health, and ability of the patient.

## CONCLUSION

The recovery of human character[4], purpose of life[44,45,46,47,48,49], coherence[53], and will to live[5,6,7,8,9,10,11,12], with consciousness-based medicine seems highly efficient in improving the quality of life of patients with metastasized cancer and presumably also their survival in some cases. We find it important that the holistic treatment, which for the patient highly motivated for personal development can be done with a limited number of hours of holistic therapy during a few days or weeks, is developed to a general offer to patients with metastasized cancer. From a holistic perspective, cancer can be understood as a simple disturbance of the cells, arising from the tissue holding on to a trauma with strong emotional content, in what we call "a blockage", allocating the function of the cells from their original function in the tissue to a function of holding emotions; improving quality of life and helping the patient to process and integrate these traumas might even help the patients to survive longer.

We believe that the consciousness-based/holistic medical toolbox has a serious offer to patients with metastasized cancer, and we will therefore strongly encourage the scientific society to explore these new possibilities. Our holistic medical research meets both ethical dilemmas and practical difficulties, but it seems evident from case studies that if the patient is motivated for personal development, quality of life can easily be improved by helping the patient recover her character, purpose of life, and will to live.

To support the patient in learning from her disease the mastery of coherence of body and life, and using the crisis of cancer to recover the human character and the purpose of life seems turning a personal potential disaster into the greatest gift of all. When it comes down to it, life is not just about surviving; what is more important is to life fully, to learn from the great challenges of life, and to obtain the optimal quality of life while being here.

## ACKNOWLEDGMENTS

We are most thankful for the kind permission to bring both the patient's case story and her diary. This study was supported by grants from IMK Almene Fond. The quality of life research was approved by the Copenhagen Scientific Ethical Committee under number (KF)V.100.2123/91.

## BIOSKETCHES

**Søren Ventegodt, MD,** is the Director of the Quality of Life Research Center in Copenhagen, Denmark. He is also responsible for a Research Clinic for Holistic Medicine in Copenhagen and is a popular speaker throughout Scandinavia. He has published numerous scientific or popular articles and a number of books on holistic medicine, quality of life, and quality of working life. His most important scientific contributions are the comprehensive SEQOL questionnaire, the very short QoL5 questionnaire, the integrated QOL theory, the holistic process theory, the life mission theory, and the Danish Quality of Life Research Survey, 1991–94 in cooperation with the University Hospital of Copenhagen and the late pediatric professor Bengt Zachau-Christiansen. E-mail: ventegodt@livskvalitet.org. Website: http://www.livskvalitet.org

**Elin Solheim** is physiotherapist, yoga teacher, and a student of holistic medicine at the Research Clinic for Holistic Medicine at the Quality of Life Research Center in Copenhagen.

**Mads E. Saunte, MD** is attending at the Research Clinic for Holistic Medicine at the Quality of Life Research Center in Copenhagen.

**Mohammed Morad, MD**, is Specialist in Family Medicine, Lecturer in Family Medicine at the National Institute of Child Health and Human Development, Division of Community Health, Ben Gurion University of the Negev and the Medical Director of a large area clinic in the city of Beer-Sheva. He has publications on Bedouin health, health aspects, spiritual health, and aging in persons with intellectual disability, and is a presenter on topics such as health policy and services for the disadvantaged at national and international conferences. E-mail: morad62@barak-online.net

**Isack Kandel**, **MA, PhD**, is Senior Lecturer at the Faculty of Social Sciences, Department of Behavioral Sciences, the Academic College of Judea and Samaria, Ariel. During the period 1985–1993, he served as the Director of the Division for Mental Retardation, Ministry of Social Affairs, Jerusalem, Israel. E-mail: Kandeli@aquanet.co.il

**Joav Merrick, MD, DMSc,** is Professor of Child Health and Human Development affiliated with the Zusman Child Development Center, Division of Pediatrics and Community Health at the Ben Gurion University, Beer-Sheva, Israel; the Medical Director of the Division for Mental Retardation, Ministry of Social Affairs, Jerusalem; and the Founder and Director of the National Institute of Child Health and Human Development. He has numerous publications in the field of child and human development, rehabilitation, intellectual disability, disability, health, welfare, abuse, advocacy, quality of life, and prevention. Dr. Merrick received the Peter Sabroe Child Award for outstanding work on behalf of Danish Children in 1985 and the International LEGO-Prize ("The Children's Nobel Prize") for an extraordinary contribution towards improvement in child welfare and well being in 1987. E-mail: jmerrick@internet-zahav.net. Website: www.nichd-israel.com
